# Comparison between the Effects of Dexmedetomidine and Midazolam on Postoperative Cognitive Impairment after Coronary Artery Bypasses Graft Surgery: A Randomized Clinical Trial 

**Published:** 2019-04

**Authors:** Mahsa Rajaei, Masoomeh Tabari, Ghassem Soltani, Kambiz Alizadeh, Alireza Nazari, Maryam Noroozian, Negar Morovatdar

**Affiliations:** 1 *Department of Anesthesiology, School of Medicine,* *Mashhad University of Medical Sciences, Mashhad, * *Iran.*; 2 *Department of Cardiac Surgery,* *School of Medicine,* *Mashhad University of Medical Sciences, Mashhad, Iran.*; 3 *Mashhad University of Medical Sciences, Mashhad, Iran.*; 4 *Department of Psychiatry, School of Medicine, Tehran University of Medical Sciences, Tehran, Iran.*; 5 *Clinical Research Unit, School of Medicine, Mashhad University of Medical Sciences, Mashhad, Iran.*

**Keywords:** *Cognitive dysfunction*, *Coronary artery bypass*, *Dexmedetomidine*, *Midazolam*

## Abstract

**Background:** Postoperative cognitive decline is a common complication observed frequently after general anesthesia in the immediate postoperative phase. We studied the effects of dexmedetomidine versus midazolam during coronary artery bypass graft (CABG) surgery on cognitive and memory function.

**Methods:** In this clinical trial, 42 elective on-pump CABG candidates under general anesthesia, aged between 40 and 65 years, were enrolled randomly in 2 groups. Group A received 0.05–0.1 mg/kg of midazolam and Group B received 1 µg/kg of dexmedetomidine. One day before surgery, all the participants underwent the Persian version of the Mini-Mental State Examination (MMSE) and the Persian version of the Wechsler Memory Scale (WMS) test for a comparison of cognitive impairment and memory functions. Both groups were given fentanyl and propofol for the induction of anesthesia and muscle relaxants. The MMSE and WMS tests were repeated 5 and 30 days after surgery.

**Results:** The mean±SD of age was 55.47±7.18 y in Group A and 55.39±6.08 y in Group B. Eighty percent of the participants were men in both groups. There were no significant differences between Group A and Group B in the MMSE and WMS before surgery (89.04±14.30 vs. 97.10±18.10, respectively; P=0.059), but the WMS was significantly different 30 days after surgery (87.60±14.30 vs. 103.53±19.93, respectively; P=0.005). Group A showed high cognitive impairment and low WMS scores compared with Group B (P=0.005). Additionally, the MMSE results were not statistically different between the 2 groups postoperatively (24.80±3.18 vs. 23.55±4.18, respectively; P=0.394).

**Conclusion:** Our results showed that dexmedetomidine might have a lower impact on cognitive function than might midazolam among patients undergoing CABG.

## Introduction

Postoperative cognitive decline or dysfunction (POCD) is a common postoperative complication that is frequently observed after general anesthesia in the immediate postoperative phase or in some cases up to 4 weeks postoperatively. POCD can persist for months or even be life-long. ^1^^, ^^2^ This problem is often transitory and may be influenced by general postoperative readjustment, opioids, pain, and fatigue.^2^ In patients undergoing general anesthesia, researchers have noted a wide range of neurocognitive impairment including stroke, postoperative delirium, depression, and early- or late-onset memory impairment.^3^ Microembolisms, hypoperfusion, risk factors for heart disease, and techniques used in surgery could result in these complications.^4^ Many of these factors such as age or genetics are unmodifiable.^5^ However, some factors such as anesthetic medications and surgical techniques can be controlled and optimized.^6^ Indeed, cognitive impairment following surgery, especially cardiac surgeries, is the result of brain ischemia and hypoperfusion that occurs during surgery for a variety of reasons. 

Regarding surgical techniques, several researchers have noted a POCD incidence rate of 53% following on-pump variants of coronary artery bypass graft (CABG) surgery.^7^ Moreover, anesthesia could also influence the incidence and severity of such complications due to the effects of anesthetic medications or potential incidents in the induction or maintenance of anesthesia.^8^ In contrast, some anesthetic medications may also have a protective effect on brain function.^9^ Opioids, midazolam, and dexmedetomidine are usually administered as sedatives for patients under monitored anesthesia care.^10^^, ^^11^ Benzodiazepines are sedative, anxiety-reducing, and hypnotic drugs that because of their effects through GABA receptors are widely used in clinical anesthesia.^12^ Midazolam, a benzodiazepine, is used immediately before the induction of anesthesia and causes loss of ability to form new memories. It, however, does not relieve pain and a high dosage of it can lead to increased recovery time, a weakened respiratory system, and decreased arterial oxygen saturation.^13^ Some studies have reported reduced cognitive impairment between 1 and 3 days after surgery when dexmedetomidine is administered. Dexmedetomidine, an α2-adrenergic receptor agonist, also has sedative, anxiolytic, hypnotic, analgesic, and sympatholytic effects. Two trials have shown that dexmedetomidine, by comparison with midazolam or propofol, improves pain tolerance in patients.^14^ It is used as a sedative and pain reliever, without having a risk of respiratory depression. Anesthetic medications can directly impact the nervous system with such clinical presentations as cognitive impairment and memory deterioration.^6^^, ^^15^^, ^^16^ Aggravated cognitive impairment can even occur in short surgical interventions, which is considered a significant issue.^7^^, ^^15^ POCD can lead to increased recovery time and negatively affect patients’ quality of life.^17^

In view of the prescription of a variety of sedative medicines and the high impact of cognitive and memory functions on quality of life, there is a critical need for the analysis of factors affecting memory and cognitive functioning postoperatively. Accordingly, in the present study, we evaluated the effects of dexmedetomidine and midazolam during CABG anesthesia on cognitive and memory function, with a view to establishing interventions aimed at reducing cognitive and memory impairment and improving the health and well-being of patients undergoing surgery.

## Methods

Forty-five patients undergoing elective CABG with general anesthesia at Ghaem and Shariati hospitals, Mashhad, Iran, between 2016 and 2018, who met the study’s inclusion criteria were enrolled in this randomized controlled trial.

The study’s proposal was registered at the Iranian Registry of Clinical Trials (IRCT) (IRCT 20170429033680N7, registration No. 33680) and was approved by the Ethics Committee of Mashhad University of Medical Sciences, Mashhad, Iran (Code: IR.MUMS.sm.REC.1394.553). 

This study was double-blind, and all the participants signed an informed written consent form for participation. Our inclusion criteria were age between 20 and 65 years and candidacy for elective CABG under general anesthesia. The exclusion criteria included lack of cooperation until the end of the trial, a history of heart attacks and long cardiopulmonary resuscitation, a history of deep vein thrombosis in the preceding 6 months, a history of advanced renal or liver diseases, surgical durations exceeding 5 hours,a history of cerebrovascular accidents, patients with no formal education, and patients with a mean arterial pressure of less than 60 mmHg for more than 2 minutes or a peripheral capillary oxygen saturation level of less than 90% for more than 1 minute during surgery. 

One day before surgery, all the participants underwent the Persian versions of the Mini-Mental State Examination (P-MMSE) and the Wechsler Memory Scale (WMS) test to determine cognitive impairment and memory functions. On the day of surgery after standard monitoring, arterial line placement was carried out. The patients were randomly assigned to the midazolam group (Group A, n=23) or the dexmedetomidine group (Group B, n=22) based on the electronically randomized method.

Group A received midazolam 0.05–0.1 mg/kg (Caspian Tamin Pharmaceutical Co., Iran) and Group B received dexmedetomidine 1 μgµg/kg (Precedex) (Hospira, Inc., Lake Forest, USA), intravenously. For both groups, fentanyl (Aburaihan Pharmaceutical Co., Iran) and propofol (Dongkook Pharm. Co., Korea) were administered for the induction of anesthesia, as well as muscle relaxants, to facilitate intubation. Additionally, fentanyl and propofol infusion was used for the maintenance of anesthesia to keep the bispectral index in the range of 40 to 60. The patients underwent on-pump beating CABG. Aortic and venous cannulation was done, without cardioplegia and aortic cross-clamping. If needed, Apotel or fentanyl was used as an analgesic after surgery.

The cognitive and memory tests were done 1 day before, and subsequently 5 and 30 days after surgery, through 2 examinations (MMSE and WMS). Then, the postoperative results of these tests were compared with the preoperative results and the results of the other group. In addition, cerebrovascular accidents and the duration of stay in the intensive care unit for each participant were recorded.

The MMSE is a cognitive impairment measurement widely used to screen for dementia and the estimation of cognitive changes over time. We used the Persian version of the examination, which consists of three 5-point items (each consisting of 5 questions), three 3-point items, one 2-point item, and four 1-point items, for a total of 30 points. The 5-point items are concerned with time and place orientation. The 3-point items comprise following 3-staged commands (e.g., stand up, walk, and sit) and delayed and immediate memory. The 2-point item is about naming 2 objects, 1 point for each. Finally, the four 1-point items consist of reading, writing, repeating a phrase, and drawing. The minimum score is 29 for individuals with higher education, 27 for individuals with secondary and high school education, 25 for individuals with elementary education, and 19 for illiterate individuals.^8^

The WMS is an objective measure for memory function and provides appropriate information regarding memory function that differentiates clinical groups. We used the Persian version of the Wechsler Memory Scale (P-WMS). This test can be used to measure learning, the recollection of immediate or delayed memory, focus, attention, and orientation. We used the WMS test consisting of 7 subsets with some modifications. The modules are comprised of: 1) Personal awareness of daily and personal routines, 2) Awareness of time and place (spatial and temporal orientation), 3) Mental control, 4) Logical memory, 5) Repeating digits forward and backward, 6) Visual memory, and 7) Symbol learning.

We calculated the sample size based on the assumption of a large effect size (d=0.8) with respect to α=0.05 and β=0.2 for the comparison of 2 independent means, using the G Power software, which estimated 22 patients per group. All the data were entered into the SPSS software, version 16 (Chicago, SPSS Inc), and analyzed. Numbers and frequencies were used for the categorical data and mean±standard deviations (SDs) for the continuous variables. The Student *t*-test or the Mann–Whitney test was applied to compare the continuous variables between the 2 groups. The χ^2 ^test was employed to compare the categorical variables between the 2 groups. ANOVA for Repeated Measures was utilized to analyze the differences between the 2 groups during the follow-up period. The Spearman correlation coefficient between “pre and post hemoglobin” and “WMS and MMSE” was calculated. A P value of less than 0.05 was considered statistically significant. 

## Results

**Figure 1:**
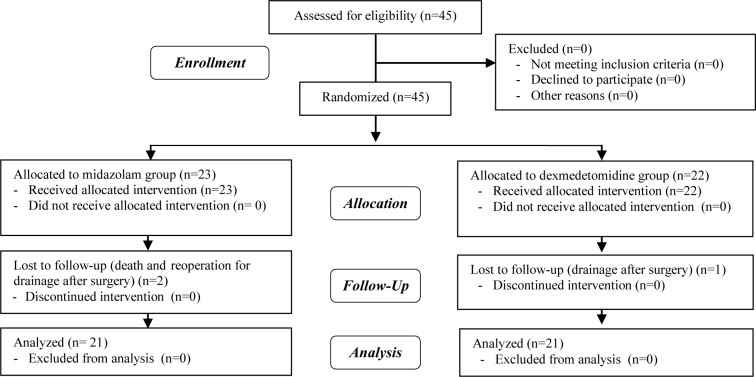
Participants’ follow-up diagram

The sociodemographic characteristics of the participants are depicted in Table 1. Overall, 45 participants in 2 groups were enrolled in the study. Two patients were excluded due to death and reoperation for drainage after surgery in Group A. In Group B, 1 patient was excluded due to drainage after surgery. Finally, 21 participants in each group were enrolled in the study (Figure 1). 

There were no significant differences between the 2 groups in the MMSE and WMS before surgery, but the WMS was significantly different during a 30-day follow-up period after surgery (P=0.026). The midazolam group showed higher cognitive impairment and lower WMS scores than did the dexmedetomidine group (Table 2). The WMS results showed that the preoperative mental control and logical memory among the participants of the 2 groups had no significant difference, while the difference was statistically significant during the 30-day follow-up period after surgery (P=0.016 and P=0.046, respectively) (Table 2).

The results of the MMSE showed that the 2 groups were not significantly different with respect to any of our tests (pre- or postoperative) (Table 3).

There was no statistically significant relationship between the hemoglobin, WMS, and MMSE results pre- or postoperatively (Table 4).

The midazolam group showed higher cognitive impairment and lower WMS scores than did the dexmedetomidine group.

**Table 1:** Demographic characteristics of the participants in the 2 study groups*

	Group A (Midazolam) (N=21)	Group B (Dexmedetomidine) (N=21)
Age (y)	55.47±7.18	55.39±6.08
Sex*		
Female	4 (17.4)	4 (18.2)
Male	19 (82.6)	18 (81.8)
History of DM	11 (47.8)	12 (54.5)
History of HTN	9 (39.1)	14 (60.0)
History of HLP	11 (47.8)	14 (66.7)
Postoperative arrhythmia	0	2 (9.5)
Need for reoperation	2 (8.7)	1 (4.5)

DM, Diabetes mellitus; HTN, Hypertension; HLP, Hyperlipidemia

* Data are presented as mean±SD or n (%).

**Table 2:** Results of the Wechsler Memory Scale (WMS) in the 2 study groups*

	Group A (Midazolam) (N=21)	Group B (Dexmedetomidine) (N=21)	P**	P***
Wechsler Memory Scale				0.026
Preoperative	89.04±14.30	97.10±18.10	0.059	
5 days after surgery	87.47±12.88	98.40±19.50	0.035	
30 days after surgery	87.60±14.30	103.53±19.93	0.005	
Detailed results of the WMS				
Personal awareness 1				0.706
Preoperative	5.52±0.81	5.23±0.74	0.373	
5 days after surgery	5.66±0.79	5.71±0.56	0.806	
30 days after surgery	6.33±1.79	6.80±2.33	0.374	
Orientation				0.621
Preoperative	4.95±0.49	4.85±0.35	0.202	
5 days after surgery	4.95±079	4.09±0.3	0.348	
30 days after surgery	4.95±0.49	4.90±0.33	0.348	
Mental control				0.003
Preoperative	5.76±2.95	7.52±1.96	0.475	
5 days after surgery	5.28±2.66	7.42±1.93	0.003	
30 days after surgery	5.04±2.45	7.71±1.64	<0.0001	
Logical memory				0.029
Preoperative	6.28±3.3	8.28±4.5	0.143	
5 days after surgery	6.71±4.01	9.50±5.31	0.046	
30 days after surgery	6.38±3.94	10.20±5.52	0.016	
Repeating digits forward and backward				0.541
Preoperative	6.52±2.04	6.76±2.36	0. 526	
5 days after surgery	6.45±2.01	6.70±2.35	0.373	
30 days after surgery	6.33±1.79	6.80±2.33	0.188	
Visual memory				0.141
Preoperative	6.19±3.15	7.33±1.95	0.340	
5 days after surgery	6.23±2.99	7.05±0.00	0.360	
30 days after surgery	6.42±2.83	7.76±1.60	0.137	
Visual reproduction				0.402
Preoperative	9.14±2.76	9.85±3.52	0.627	
5 days after surgery	9.14±3.05	9.61±3.33	0.713	
30 days after surgery	8.95±2.67	10.04±3.10	0.130	

* Data are presented as mean±SD.

** Mann–Whitney test

*** Repeated measures of ANOVA

**Table 3:** Results of the Mini-Mental State examination in the 2 study groups*

	Group A (Midazolam) (N=21)	Group B (Dexmedetomidine) (N=21)	P**	P***
Mini-Mental State Score				0.124
Preoperative	25.57±3.41	22.61±5.60	0.108	
5 days after surgery	24.76±3.16	22.40±4.90	0.120	
30 days after surgery	24.80±3.18	23.55±4.18	0.394	

* Data are presented as mean±SD.

** Mann–Whitney test

*** Repeated measures of ANOVA

**Table 4:** Relationships between hemoglobin, the Wechsler Memory Scale (WMS), and the Mini-Mental State Examination (MMSE) at various time points

		Preoperative Hemoglobin	Postoperative Hemoglobin
Wechsler Memory Scale			
Preoperative	r	0.167	0.216
	P	0.297	0.175
5 days after surgery	r	0.149	0.230
	P	0.353	0.147
30 days after surgery	r	0.228	0.270
	P	0.151	0.088
Mini-Mental State Examination			
Preoperative	r	-0.072	0.013
	P	0.656	0.934
5 days after surgery	r	-0.010	0.060
	P	0.952	0.710
30 days after surgery	r	0.000	0.040
	P	0.998	0.804

r, Spearrman correlation coefficient test; P, P value

## Discussion

Our analysis of memory and cognitive impairment showed that the 2 study groups were significantly different with respect to the WMS postoperatively. The dexmedetomidine group showed fewer signs of cognitive impairment than did the midazolam group, 5 and 30 days after surgery. In addition, the mental control item and the logical memory item of the test were significantly better in the dexmedetomidine group than in the midazolam group. 

Other studies have reported similar results. In China, Qian and co-workers^   18 ^ examined the effects of dexmedetomidine on POCD. The goal of their study was to identify how dexmedetomidine protects the brain after major surgery in middle-aged patients. The researchers measured cognitive impairment using the Y-Maze method. They also measured inflammatory cytokine interleukin 1 beta, tumor necrosis factor, and apoptosis-related factor caspase-3 via polymerase chain reaction and analyzed the findings using western blot. Their results showed that the administration of dexmedetomidine significantly improved cognitive function on the first and third days after surgery. In addition, there was an increase in inflammatory cytokines following surgery, which went down remarkably with the use of dexmedetomidine. Furthermore, they reported that POCD could contribute to hippocampal inflammatory response and neuronal apoptosis and that selective alpha 2 adrenal receptor excitation could play a protective role. This is in line with our findings insofar as we found significantly lower cognitive impairment in the dexmedetomidine group 5 and 30 days after surgery compared with the midazolam group. 

Maldonad and colleagues^   19 ^ investigated the effects of dexmedetomidine on the reduction of postoperative delirium following CABG on patients who underwent elective cardiac surgery with general anesthesia and were then randomly assigned to 1 of 3 sedative postoperative protocols: dexmedetomidine, propofol, or midazolam. Their results showed that the incidence of delirium was 3% in the dexmedetomidine group, 50% in the propofol group, and 50% in the midazolam group. This showed the significantly lower incidence of delirium in the dexmedetomidine group by comparison with the others. Their findings also showed that dexmedetomidine administration postoperatively significantly reduced back pain after surgery and also reduced care costs. In another study, the effects of dexmedetomidine and midazolam on human cognitive function and mood were analyzed. The results indicated that dexmedetomidine, depending on the dosage administered, could lead to some visual and mental impairment. In addition, the authors of that investigation asserted that the effects of dexmedetomidine might primarily impact the cardiovascular system by controlling the sympathetic nervous system and that sedation was a secondary effect achieved with higher doses of dexmedetomidine. Even though the mental effects of dexmedetomidine appeared quickly, cognitive impairment and, importantly, the decrease in systolic and diastolic blood pressures appeared over time. It was also observed that both of them had similar quantitative and qualitative effects on the pain.^   20 ^ Bisetto and others^   21 ^ examined the sedative and analgesic effects of dexmedetomidine, midazolam, and dexmedetomidine–midazolam mix in a group of 6 tegus (Salvator Merianae). The results showed that midazolam had sedative, but not analgesic, effects. In contrast, dexmedetomidine had analgesic, but not sedative, effects. The combination of these 2 increased the sedative effects but failed to result in increased analgesic effects. Arpaci et al.^   22 ^ performed a comparison of the effectiveness of remifentanil-dexmedetomidine and remifentanil-midazolam combinations for patients under monitored anesthesia care for cystoscopies. The results showed that target sedation levels were achieved faster in the dexmedetomidine group than in the midazolam group (P<0.0001). Dexmedetomidine also had remarkably less influence on cognitive function than did midazolam (P<0.0001). In addition, the dexmedetomidine group had notably higher satisfaction levels among both patients and surgeons. 

We also found that the relationship between cognitive impairment and the medication had no correlation with hemoglobin levels. The hematocrit value may be related to the outcome after CABG. Habib and associates^   23 ^ found that the lowest quintile of hematocrit was related to vital organ dysfunction, morbidity, and 6-year survival in a survey of 5000 consecutive operations. Also, Karkouti and colleagues^24^ found a positive association between the risk of perioperative stroke and the lowest operative hematocrit. However, in a study examining cognitive dysfunction, Harrison and associates^   25 ^ found no correlation between cognitive outcome and hematocrit.

Further studies in this area are strongly encouraged. There are some limitations to our study. Firstly, due to the repetition of the same cognitive tests, the patients might have learned and memorized the questions; thus, the results for the subsequent tests might have been inflated (practice effect). Secondly, one day before surgery, the patients are usually under a great deal of stress and anxiety, which negatively affects their cognitive tests. In contrast, 30 days after surgery, having gained confidence in their well-being and surgery outcome, patients may enjoy an improvement in their cognitive function.

To the best of our knowledge, this is the first study to compare the effects of midazolam and dexmedetomidine on POCD in on-pump beating CABG in Iran. 

## Conclusion

Our results showed that dexmedetomidine had a lower impact on cognitive function than did midazolam. The results of this study support the administration of dexmedetomidine instead of midazolam in cardiac surgery.

## Notes:

***This paper should be cited as: ***Rajaei M, Tabari M, Soltani G, Alizadeh K, Nazari A, Noroozian M, Morovatdar N. Comparison between the Effects of Dexmedetomidine and Midazolam on Postoperative Cognitive Impairment after Coronary Artery Bypasses Graft Surgery: A Randomized Clinical Trial. J Teh Univ Heart Ctr 2019;14(2):67-73.

## References

[1] Chen PL, Yang CW, Tseng YK, Sun WZ, Wang JL, Wang SJ, Oyang YJ, Fuh JL (2014). Risk of dementia after anaesthesia and surgery. Br J Psychiatry.

[2] Baumgartner WA (2007). Neurocognitive changes after coronary bypass surgery. Circulation.

[3] Xie Z, Dong Y, Maeda U, Moir R, Inouye SK, Culley DJ, Crosby G, Tanzi RE (2006). Isoflurane-induced apoptosis: a potential pathogenic link between delirium and dementia. J Gerontol A Biol Sci Med Sci.

[4] Selnes OA, Pham L, Zeger S, McKhann GM (2006). Defining cognitive change after CABG: decline versus normal variability. Ann Thorac Surg.

[5] Newman MF, Kirchner JL, Phillips-Bute B, Gaver V, Grocott H, Jones RH, Mark DB, Reves JG, Blumenthal JA, Neurological Outcome Research Group and the Cardiothoracic Anesthesiology Research Endeavors Investigators (2001). Longitudinal assessment of neurocognitive function after coronary-artery bypass surgery. N Engl J Med.

[6] Mashour GA, Forman SA, Campagna JA (2005). Mechanisms of general anesthesia: from molecules to mind. Best Pract Res Clin Anaesthesiol.

[7] Tzabar Y, Asbury AJ, Millar K (1996). Cognitive failures after general anaesthesia for day-case surgery. Br J Anaesth.

[8] Evered L, Scott DA, Silbert B, Maruff P (2011). Postoperative cognitive dysfunction is independent of type of surgery and anesthetic. Anesth Analg.

[9] Hope At, Woolman PS, Gray WM, Asbury AJ, Millar K (1998). A system for psychomotor evaluation; design, implementation and practice effects in volunteers. Anaesthesia.

[10] Froehlich F, Schwizer W, Thorens J, Köhler M, Gonvers JJ, Fried M (1995). Conscious sedation for gastroscopy: patient tolerance and cardiorespiratory parameters. Gastroenterology.

[11] Arrowsmith JB, Gerstman BB, Fleischer DE, Benjamin SB (1991). Results from the American Society for Gastrointestinal Endoscopy/U.S. Food and Drug Administration collaborative study on complication rates and drug use during gastrointestinal endoscopy. Gastrointest Endosc.

[12] Winsky-Sommerer R (2009). Role of GABAA receptors in the physiology and pharmacology of sleep. Eur J Neurosci.

[13] Fassoulaki A, Theodoraki K, Melemeni A (2010). Pharmacology of sedation agents and reversal agents. Digestion.

[14] Maze M, Tranquilli W (1991). Alpha-2 adrenoceptor agonists: defining the role in clinical anesthesia. Anesthesiology.

[15] Hanning CD (2005). Postoperative cognitive dysfunction. Br J Anaesth.

[16] Heinke W, Koelsch S (2005). The effects of anesthetics on brain activity and cognitive function. Curr Opin Anaesthesiol.

[17] Phillips-Bute B, Mathew JP, Blumenthal JA, Grocott HP, Laskowitz DT, Jones RH, Mark DB, Newman MF (2006). Association of neurocognitive function and quality of life 1 year after coronary artery bypass graft (CABG) surgery. Psychosom Med.

[18] Qian XL, Zhang W, Liu MZ, Zhou YB, Zhang JM, Han L, Peng YM, Jiang JH, Wang QD (2015). Dexmedetomidine improves early postoperative cognitive dysfunction in aged mice. Eur J Pharmacol.

[19] Maldonado JR, Wysong A, van der Starre PJ, Block T, Miller C, Reitz BA (2009). Dexmedetomidine and the reduction of postoperative delirium after cardiac surgery. Psychosomatics.

[20] Mattila MJ, Mattila ME, Olkkola KT, Scheinin H (1991). Effect of dexmedetomidine and midazolam on human performance and mood. Eur J Clin Pharmacol.

[21] Bisetto SP, Melo CF, Carregaro AB (2018). Evaluation of sedative and antinociceptive effects of dexmedetomidine, midazolam and dexmedetomidine-midazolam in tegus (Salvator merianae). Vet Anaesth Analg.

[22] Arpaci AH, Bozkırlı F (2013). Comparison of sedation effectiveness of remifentanil-dexmedetomidine and remifentanil-midazolam combinations and their effects on postoperative cognitive functions in cystoscopies: A randomized clinical trial. J Res Med Sci.

[23] Habib RH, Zacharias A, Schwann TA, Riordan CJ, Durham SJ, Shah A (2003). Adverse effects of low hematocrit during cardiopulmonary bypass in the adult: should current practice be changed?. J Thorac Cardiovasc Surg.

[24] Karkouti K, Djaiani G, Borger MA, Beattie WS, Fedorko L, Wijeysundera D, Ivanov J, Karski J (2005). Low hematocrit during cardiopulmonary bypass is associated with increased risk of perioperative stroke in cardiac surgery. Ann Thorac Surg.

[25] Harrison MJ, Stygall J, Whitaker DC, Grundy NM, Newman SP (2006). Hematocrit during cardiopulmonary bypass. Ann Thorac Surg.

